# Prevalence of Tourette Syndrome and Chronic Tics in the Population-Based Avon Longitudinal Study of Parents and Children Cohort

**DOI:** 10.1016/j.jaac.2011.11.004

**Published:** 2012-02

**Authors:** Jeremiah M. Scharf, Laura L. Miller, Carol A. Mathews, Yoav Ben-Shlomo

**Affiliations:** a Psychiatric and Neurodevelopmental Genetics Unit, Center for Human Genetics Research, Massachusetts General Hospital, and the Division of Cognitive and Behavioral Neurology, Brigham and Women's Hospital; b School of Social and Community Medicine, University of Bristol, UK; c University of California at San Francisco

**Keywords:** Tourette syndrome, prevalence, ALSPAC, obsessive-compulsive disorder, attention-deficit/hyperactivity disorder

## Abstract

**Objective:**

Recent epidemiologic studies have demonstrated that Tourette syndrome (TS) and chronic tic disorder (CT) are more common than previously recognized. However, few population-based studies have examined the prevalence of co-occurring neuropsychiatric conditions such as obsessive-compulsive disorder (OCD) and attention-deficit/hyperactivity disorder (ADHD). We evaluated the prevalence of TS, CT, and their overlap with OCD and ADHD in the Avon Longitudinal Study of Parents and Children (ALSPAC) birth cohort.

**Method:**

A total of 6,768 children were evaluated using longitudinal data from mother-completed questionnaires. *DSM-IV-TR* diagnoses of TS and CT were derived using three levels of diagnostic stringency (Narrow, Intermediate, and Broad). Validity of the case definitions was assessed by comparing gender ratios and rates of co-occurring OCD and ADHD using heterogeneity analyses.

**Results:**

Age 13 prevalence rates for TS (0.3% for Narrow; 0.7% for Intermediate) and CT (0.5% for Narrow; 1.1% for Intermediate) were consistent with rates from other population-based studies. Rates of co-occurring OCD and ADHD were higher in TS and CT Narrow and Intermediate groups compared with controls but lower than has been previously reported. Only 8.2% of TS Intermediate cases had both OCD and ADHD; 69% of TS Intermediate cases did not have either co-occurring OCD or ADHD.

**Conclusions:**

This study suggests that co-occurring OCD and ADHD is markedly lower in TS cases derived from population-based samples than has been reported in clinically ascertained TS cases. Further examination of the range of co-occurring neuropsychiatric disorders in population-based TS samples may shed new perspective on the underlying shared pathophysiology of these three neurodevelopmental conditions.

Tourette syndrome (TS) is a chronic, childhood-onset neuropsychiatric disorder characterized by waxing and waning motor and vocal tics that persist for more than 1 year.^1^ Tics usually begin between 5 and 7 years of age, are most severe in early adolescence, and then gradually decrease in early adulthood.^2,3^ Chronic tic disorder (CT), which is defined by the presence of either motor or vocal tics (but not both), is similar in clinical phenomenology and disease course to TS, but is less frequently associated with co-occurring neuropsychiatric conditions, such as obsessive-compulsive disorder (OCD) and attention-deficit/hyperactivity disorder (ADHD).^4^ TS and CT cause significant physical and psychosocial morbidity, and in severe cases can produce lifelong disability.^2,5^

TS was initially considered to be rare, with early estimates of approximately 5 per 10,000 school-age children (0.05%).^6^ However, these studies included only clinically ascertained cases, an approach that greatly underestimates the true prevalence of the disorder by excluding individuals who do not seek treatment. In contrast, a number of population-based studies have been conducted over the past two decades that suggest that TS is much more common, with most prevalence estimates converging around a rate of 0.3% to 0.8% of the school-age population.^7,8^ Fewer studies have examined the prevalence of CT, and estimates range from 1.3% to 3.7% of children.^9-11^ Determination of accurate TS/CT prevalence estimates is important for assessing the overall burden of disease, allocating treatment resources, and estimating the familial risk in relatives of TS patients.

In addition, TS and CT are frequently associated with multiple co-occurring neuropsychiatric conditions in clinically ascertained samples, particularly OCD and ADHD. In the largest clinical study of 3,500 TS patients from 64 international clinics, OCD was present in 27% (range 2%–66%), whereas 60% had ADHD (range 33%–91%).^12^ Furthermore, only 12% of TS patients (range 2%–35%) had tics without any other co-existing disorders.^12^ A more recent U.S. telephone-based survey of clinician-diagnosed TS found that 64% of children with TS had ADHD and 79% had at least one co-occurring neuropsychiatric condition.^13^ Although these clinic-based estimates are important for informing clinical practice, they may overestimate the true rates of co-occurring disorders with TS in the general population because of referral bias. Various studies have examined rates of TS-related OCD and/or ADHD in the general population.^4,14-20^ Some of these studies suggest that community-based TS subjects have lower rates of OCD^4,17,19^ (0%–19%) and ADHD^15^ (8%) than cases ascertained through clinics, although others are consistent with rates found in clinical populations (42% for OCD^15^ and 36%–100% for ADHD).^4,14,16,17,19,20^ In addition, only one of these population-based studies examined concurrent OCD and ADHD in CT specifically,^4^ although other studies have examined rates of ADHD across the tic spectrum.^7,21^ A more comprehensive understanding of the relationship and overlap between TS/CT, OCD, and ADHD in the general population would provide a framework for studies of the underlying genetics and pathophysiology of these disorders.

Here, we determined the prevalence of TS and CT, as well as the rates of co-occurring OCD and ADHD, in the Avon Longitudinal Study of Parents and Children (ALSPAC) sample, an ongoing, prospective, population-based birth cohort study in which tic, OCD, and ADHD symptoms were assessed by maternal screening questionnaires at multiple time points throughout childhood.

## Method

### Subjects

A total of 14,541 pregnant women resident in Avon, United Kingdom, with expected delivery dates between April 1, 1991, and December 31, 1992, were enrolled in ALSPAC, representing 85% of the eligible population.^22,23^ Of the 14,472 pregnancies with known birth outcomes, 13,988 infants were alive at 1 year. Mothers completed self-administered questionnaires about themselves and their child's development, environmental exposures, and health outcomes approximately every 6 months from birth to age 7 years and every year thereafter, with data available for 7,152 children at age 13. 99% of children were between 13 years 1 month and 13 years 11 months of age at the time the age 13 questionnaire was answered (full range: 12 years 10 months to 16 years 1 month). Ethical approval for the study was obtained from the ALSPAC Law and Ethics Committee and Local Research Ethics Committees. The characteristics of this population-based sample and its generalizability have been previously reported.^22^ Briefly, children in Avon had parents with a similar racial distribution as the general UK population (5.1% versus 6.4% nonwhite, in Avon and the entire United Kingdom, respectively), level of education (14.0 % vs 13.7% with university degrees), and the rate of single parent households at age 5 (4% versus 5%), although children in Avon were significantly less likely to have a father working in manual labor (51.6% versus 65.1%).

### Disease Definitions

ALSPAC children were evaluated for the presence of a tic disorder in nine mother-completed questionnaires from age 1.5 to 13 years (questionnaires are available at the ALSPAC Web site at http://www.bristol.ac.uk/alspac/sci-com/quests/). At yearly intervals from age 1.5 to 7.5 years and at age 10, mothers were asked a single screening question about the presence and frequency of “tics or twitches” in their child. Rates of positive response to this single tic question at each age are provided online (Table S1, available online). At age 13 years, a more detailed tic assessment was administered, including a section with five questions about specific motor and vocal tics: (C1: In the past year, has your child had any repeated movements of parts of the face and head (e.g., eye blinking, grimacing, sticking tongue out, licking lips, spitting)?; C2: Has your child had repeated movements of the neck, shoulder or trunk (e.g., twisting around, shoulder shrugging, bending over, nodding)?); C3: Has your child had repeated movements of arms, hands, legs, feet?; C4: Has your child had repeated noises and sounds (e.g., coughing, clearing throat, grunting, gurgling, hissing)? C5: Has your child had repeated words or phrases?). Each question was answered as “definitely”, “probably” or “not at all” present. An additional item queried the frequency of the repeated movements.

Diagnoses of TS and CT were defined by applying DSM-IV-TR criteria to the questionnaire responses based on three levels of stringency (Narrow, Intermediate, and Broad) (Table 1). Positive responses regarding the presence and frequency of specific motor and/or vocal tics in the Age 13 questionnaire were required for all definitions (Table 1). Positive responses at an additional time point between ages 1.5 and 10 years were required to meet *DSM-IV-TR* chronicity criteria of tic persistence for more than 1 year for the Narrow and Intermediate definitions. No chronicity criteria were required for the TS Broad definition. All assessments were performed before age 18 and thus met *DSM-IV-TR* age of onset criteria. Subjects who endorsed only repeated movements of the arms, hands, legs or feet (Question C3) or repeated words or phrases (Question C5) in the absence of a positive response to other tic questions (C1, C2, C4) were excluded from all case definitions to remove non-tic movements such as stereotypy or isolated echolalia. Response rates to each of the age 13 tic-related questions are provided online (Table S2, available online).

**FIGURE 1:**
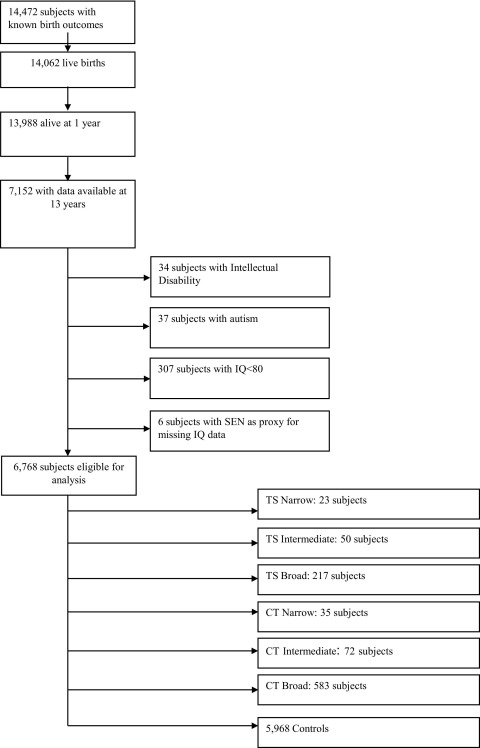
Study flow diagram. CT = chronic tics; SEN = Special Educational Needs statement; TS = Tourette syndrome.

Subjects with intellectual disability (ID) or autism were excluded to remove individuals with perseverative behaviors and stereotypies that might mimic tics. Autism and ID were defined based on a review of medical and school records as described previously.^24^ As record-review data for ID were only available for a subset of ∼900 subjects, ID was also defined based on the results of age-appropriate standard neuropsychological assessments administered at ages 4 and 8 (Wisconsin Preschool and Primary Scale of Intelligence [WPPSI] and WISC-IV, respectively). Age 4 data were examined only if age 8 data were unavailable. Subjects with full-scale IQ ≤80 were excluded. When IQ data were not available at either age (n = 1,437), the presence of a Special Educational Needs (SEN) statement for any reason other than “sensory or physical needs” was used as a proxy for low IQ; as a result, an additional six subjects were excluded (Figure 1). A total of 267 subjects had no IQ, autism, ID, or SEN statement data available. These subjects were no more likely to receive subsequent tic diagnoses compared with subjects in the main sample and thus were included in the overall analysis (data not shown). Controls were defined as subjects who were eligible for analysis (data available at age 13 and not excluded based on the presence of ID, autism, IQ <80 or an SEN statement in the absence of available IQ data), but did not meet any of the tic case definitions.

Lifetime diagnoses of *DSM-IV-TR* OCD and ADHD were derived using a self-report version of the Development and Well Being Assessment (DAWBA) parent interview that was completed by ALSPAC mothers about their children as part of the age 7, 10, and 14 questionnaires.^25^ The presence of recurrent obsessions or compulsions (response of “sometimes” or “often” to 1 or more of 7 questions about contamination, cleaning, checking, repeating, touching, arranging, or counting symptoms) that were severe enough either to last >1 hour a day, “waste a lot of time,” cause significant distress (“upset a great deal”), or cause interference or impairment (answers of “Quite a lot” or “A great deal” to five questions about interference with family, friends, school, or hobbies) at one of the three time points was required for a diagnosis of OCD. Recognition of these thoughts as excessive or unreasonable was not required per *DSM-IV-TR* guidelines for diagnosing OCD in children. Similarly, the presence of six of nine inattentive and/or six of nine hyperactive/impulsive symptoms, starting before age 7 years and causing interference in at least two of four settings (family, friends, school, leisure activities) were required to meet criteria for ADHD.

### Statistical Analyses

All statistical analyses were performed in Stata v.11. The Poisson option was used to calculate exact confidence intervals for prevalence estimates. Gender ratios and rates of co-occurring OCD and ADHD in subjects with TS and CT were compared with unaffected controls using χ^2^ statistics with Yates' adjustment for small sample sizes; exact confidence intervals were also calculated. Heterogeneity analyses between different TS/CT disease definitions were performed using Cochran's Q and I^2^ statistics. Because these TS/CT disease definitions are nested, heterogeneity was examined by comparing subjects in the more narrowly defined group (for example, TS Narrow) to the additional subjects which comprised the broader definition (e.g., TS Intermediate subjects excluding those who met criteria for TS Narrow). All statistical tests were two-tailed.

## Results

### TS and CT Prevalence Rates in the ALSPAC Cohort

Figure 1 documents the flow of subjects through the study. Of 14,472 subjects with known birth outcomes, mothers of 7,152 subjects completed the age 13 questionnaire containing the detailed tic-related questions. Of these, 384 were excluded for ID, autism, IQ<80, or an SEN statement as described above, leaving 6,768 subjects for analysis (Figure 1). Point prevalence estimates of TS and CT at age 13 were calculated for the Narrow, Intermediate, and Broad definitions (Table 2). The prevalence rates for TS Narrow and TS Intermediate (0.3% and 0.7%, respectively), as well as CT Narrow and CT Intermediate (0.5% and 1.1%) were consistent with those reported in previous community-based samples.^7,8^ In contrast, the TS Broad and CT Broad prevalence estimates (3.2% and 8.6%, respectively), which did not require that tics be chronic, were significantly higher than would be expected based on prior studies; for this reason, only the TS/CT Narrow and Intermediate definitions were included in subsequent analyses.

CT Narrow and Intermediate cases were also subdivided into those with chronic motor tics only (CMT) and chronic vocal tics only (CVT). The prevalence rates of CMT and CVT Narrow were 0.3% and 0.2%, respectively, whereas CMT and CVT Intermediate were 0.7% and 0.4% (Table S3, available online).

### Gender Ratios and Rates of Co-occurring OCD and ADHD in TS/CT Definitions

To assess the validity of the TS/CT Narrow and Intermediate disease definitions, gender ratios and rates of OCD and ADHD were compared across different TS/CT disease groups and tested formally for heterogeneity (Table 3). As expected, male-to-female gender ratios were significantly higher in both TS case definitions (TS Narrow, 3.6:1; TS Intermediate, 2.3:1) compared with controls (0.9:1; *p* = .006 and *p* = .002, respectively). A low-to-moderate degree of heterogeneity^26^ was present between the two TS definitions (I^2^ = 32.8%, *p* = .22). Similarly, CT Narrow and CT Intermediate groups had a higher proportion of males than controls (CT Narrow, 1.9:1; CT Intermediate, 2.4:1; *p* = .047 and *p* < .001, respectively) with no heterogeneity between the two CT definitions (I^2^ = 0%, *p* = .64). The gender ratios for the CMT and CVT case definitions were similar to those for overall CT with no heterogeneity between the Narrow and Intermediate groups (Table S3, available online).

Rates of OCD and ADHD were elevated in all four TS/CT disease definitions relative to controls (Table 3). OCD was present in 22% of TS Narrow and 20% of TS Intermediate subjects compared with 2% of controls (*p* < .001 for both groups), whereas 9% of CT Narrow and 10% of CT Intermediate subjects had OCD (*p* = .039 and *p* < .001, respectively). The frequency of ADHD was 17% in TS Narrow and 18% in TS Intermediate, 14% in CT Narrow and 11% in CT Intermediate, compared with 2% in controls (*p* < .001 for all comparisons). There was no evidence of heterogeneity between the Narrow and Intermediate definitions for either TS or CT with respect to OCD or ADHD (I^2^ = 0% to 2.5%). Rates of OCD and ADHD were also elevated compared with controls in the CMT Narrow/Intermediate and CVT Narrow/Intermediate groups, with a slightly higher rate of OCD in CVT Narrow and Intermediate (17% and 12%, respectively) relative to CMT Narrow (4%) and CMT Intermediate (9%) (Table S3, available online). Rates of co-occurring ADHD were 13% in both CMT definitions; the rate of ADHD was higher in CVT Narrow (17%) compared with CMT, but lower in CVT Intermediate (8%) (Table S3, available online).

**FIGURE S1:**
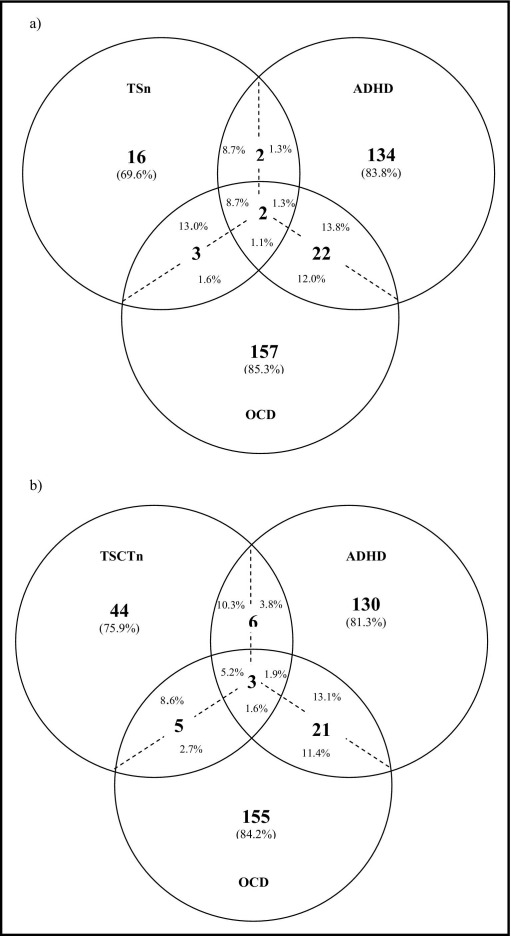
Overlap of Tourette syndrome (TS)/chronic tics (CT), obsessive-compulsive disorder (OCD) and attention-deficit/hyperactivity disorder (ADHD) diagnoses in the Avon Longitudinal Study of Parents and Children (ALSPAC) using the TS and CT narrow case definitions. (a) TS Narrow (TSn). Comparison of overlap between TS, OCD, and ADHD cases using the TSn case definition. Note: Percentages indicate the fraction of subjects in each subgroup. Percentages on either side of the dotted line indicate the different fractional percent of individuals with overlapping (co-occurring) conditions relative to the disorder of reference. For example, the two cases of TS+OCD+ADHD at the center of the diagram represent 8.7% of the total TS sample, but only 1.1% of the OCD and 1.3% of the ADHD samples, respectively. (b) TS or CT Narrow (TSCTn). Comparison of overlapping conditions between any chronic tic disorder (TS or CT), OCD, and ADHD.

**FIGURE 2:**
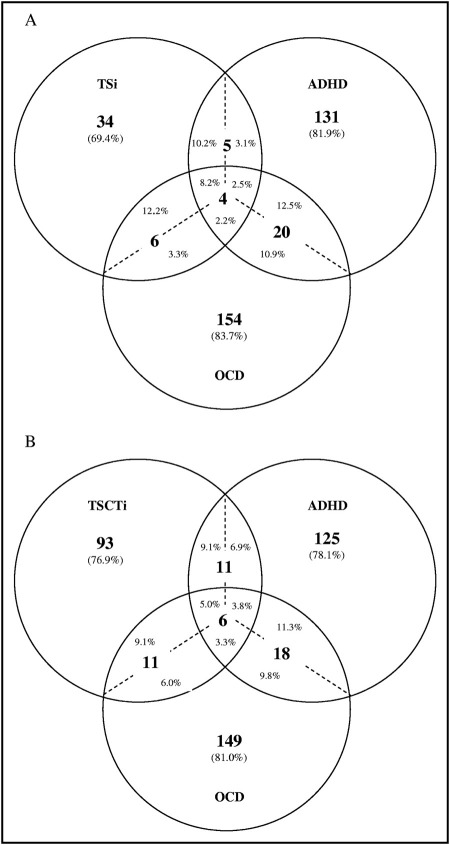
Overlap of Tourette syndrome (TS)/chronic tics (CT), obsessive-compulsive disorder (OCD) and attention-deficit/hyperactivity disorder (ADHD) diagnoses in the Avon Longitudinal Study of Parents and Children. (a) Comparison of overlap among TS, OCD, and ADHD cases using the TS Intermediate (TSi) definition. Note: Percentages indicate the fraction of subjects in each subgroup. Percentages on either side of the dotted line indicate the different fractional percent of individuals with overlapping conditions relative to the disorder of reference. For example, the five cases of TS+ADHD without OCD represent 10.2% of the total TS sample but only 3.1% of the ADHD sample. Similarly, the four TS+OCD+ADHD cases at the center of the diagram represent 8.2% of the TS sample but only 2.2% of the OCD and 2.5% of the ADHD samples, respectively. (b) Comparison of overlapping conditions between any chronic tic disorder (TS or CT), OCD and ADHD using the Intermediate case definitions of TS or CT (TSCTi).

The overlap between TS/CT, OCD and ADHD were also examined for all subjects who completed both the tic and OCD/ADHD questionnaires (n = 6,607) (Figure 2). 8.2% of TS Intermediate cases had both ADHD and OCD, whereas only 2.2% of all OCD cases and 2.5% of all ADHD cases had all three disorders (Figure 2a). In addition, 69% of TS Intermediate cases and approximately 80% of all OCD or ADHD cases had an isolated disorder without either of the other two diagnoses. This relatively low rate of overlapping TS, OCD, and ADHD was present even when considering all chronic tic disorders (TS or CT Intermediate) or when restricting the sample to the more stringent TS Narrow definition (Figure 2b and Figure S1, available online).

### Sensitivity Analysis of Tic Frequency Criterion

To assess the effect of applying strict *DSM-IV-TR* frequency criteria requiring that tics be present daily or nearly every day, a sensitivity analysis was conducted to relax the frequency criterion in TS/CT Intermediate to include children with tics occurring “about once a week.” This analysis increased the TS and CT Intermediate sample by 10% (five TS and seven CT cases), but resulted in no substantial change in prevalence, gender ratios, or rates of co-occurring OCD or ADHD (Table S4, available online).

### Examination of Attrition Bias

Because parents of children who left the study before age 13 years consistently endorsed higher rates of tics than those who remained at age 13 (Table S1, available online), we examined the factors related to attrition in the sample (Table S5, available online). Female gender, nonwhite ethnicity, lower maternal age, and markers of lower socio-economic status such as housing tenure and maternal education were all associated with loss to follow-up before age 13.

## Discussion

This study examined the point prevalence of TS and CT as well as rates of co-occurring OCD and ADHD in the population-based ALSPAC birth cohort. Both the TS Narrow and TS Intermediate definitions produced prevalence estimates (0.3% and 0.7%, respectively) that fall within the range of 0.3% to 0.8% reported by most population-based TS prevalence studies of school-age children over the past decade.^8,10,11,17,18,20^ Although some recent studies reported significantly higher TS rates (3%^27^ and 3.8%^28^), these studies were confounded by small sample size and low participation rates, respectively.^7^ Similarly, the only prior population-based study to report a markedly lower TS prevalence rate (0.04%) assessed subjects in late adolescence when tics often diminish or disappear.^2,15^ Thus, the rates observed in the current study, combined with the minimal heterogeneity observed between TS Narrow and TS Intermediate, suggest that either definition could serve as a reasonable proxy for TS in future studies.

In contrast, the prevalence estimates for CT Narrow (0.5%) and CT Intermediate (1.1%) were somewhat lower than the rates of 1.3% to 3.7% reported in prior population-based studies.^9-11^ Both CT definitions had lower male-to-female ratios and rates of co-occurring OCD and ADHD compared with the TS groups (Table 3), a finding that is consistent with the one previous population-based study that examined rates of co-occurring conditions in both TS and CT in the same cohort.^4^ In that study, most of the CT-associated OCD and ADHD arose from subjects with chronic vocal tics (CVT) (8% with OCD, 33% with ADHD) rather than subjects with chronic motor tics (CMT) (0% with OCD and 12% with ADHD). Although a trend toward higher rates of OCD was observed in the ALSPAC sample in CVT relative to CMT, the small sample size of these subgroups, particularly in the Narrow definitions, limit the interpretability of these results (Table S3, available online).

The rates of concurrent TS and OCD in the current study are consistent with those of two prior, school-based studies that identified OCD in 16%^19^ and 19%^4^ of children with TS. A third, smaller school-based study identified only seven children with TS, none of whom had OCD, placing their point estimate of co-occurring OCD at an upper limit of 14% (<1 in seven).^17^ Together, these data suggest that co-occurring OCD is less common in TS cases derived from population-based studies compared with those from clinically ascertained samples. Although one community-based study of 17-year-old Israeli army recruits identified OCD in 42% of TS subjects,^15^ this rate is not necessarily comparable to those in other studies, as OCD may be more prevalent in adolescent TS patients compared with school-age children.^3,29^

The rate of co-occurring ADHD in the ALSPAC TS sample is substantially lower than those reported in other population-based studies (36%–100%),^4,14,17,19,20,30^ although it is higher than the 8% ADHD rate reported in the Israeli TS study.^15^ This finding may be attributable to the instrument used to diagnose ADHD in ALSPAC, as previous studies have demonstrated that the DAWBA parent-form alone underestimates the true rate of ADHD in the population.^25,31^ Thus, our estimate of co-occurring ADHD should be considered a minimum prevalence in this sample.

Our study also offers the opportunity to examine the rates of overlap among TS, OCD and ADHD cases, a comparison that has not been previously reported in a population-based sample (Figure 2 and Figure S1, available online). Only 8% to 9% of ALSPAC TS cases had all three disorders (TS+OCD+ADHD) compared with 18% to 34% of clinically ascertained TS patients.^29,32^ Similarly, although fewer than 30% of TS clinic patients have TS without co-occurring OCD or ADHD,^29,32^ nearly 70% of ALSPAC TS cases did not have either of these two major co-existing conditions. These data suggest that TS individuals in the general population, compared with those seen in specialty clinics, may be more likely to have an isolated tic disorder without OCD or ADHD. This observation, if validated in future studies, would be important for community psychiatrists and pediatricians to consider when counseling patients with new diagnoses and their families. However, it is important to note that many other neuropsychiatric conditions can be associated with TS that were not examined in this study. Other population-based studies have reported high rates of disruptive behaviors in children with tics,^21,33^ and a recent population-based study found that 92% of community-based TS cases had at least one additional neuropsychiatric condition including OCD, ADHD, depression, conduct disorder, developmental coordination disorder, learning disability, sleep disorder, or mental retardation.^4^

We have taken a number of steps to minimize the impact of the study's primary limitations, the likely bias in our prevalence estimates resulting from differential attrition, and the reliance on maternal questionnaires rather than direct clinical assessments. With regard to attrition bias, it is important to note that any cross-sectional study in a school-age population inherently represents a biased sample of children compared with their original “birth cohort,” although in most cases the attrition rate is unmeasured. Here, because ALSPAC is a longitudinal study, we were able to examine the factors related to attrition and take note of potential bias, turning an inherent weakness into a strength of the study. Although the identified attrition bias might reduce the generalizability of our findings to some degree, the factors that we identified as being associated with attrition (female gender, nonwhite ethnicity, lower maternal age, and lower socio-economic status) are likely to predict nonparticipation in most epidemiologic studies. We may also have missed some subjects who had chronic tics that abated before age 13, the time point for which we have the most reliable tic-related data; however, because TS generally begins early in childhood and peaks in early adolescence, we believe that we have successfully captured most subjects.^2^ In addition, we deliberately chose rigorous disease definitions and sought to exclude subjects with likely non-tic movement disorders (e.g., stereotypies in autism or intellectual disability, repetitive arm/leg movements that could be better explained by tremor or motor restlessness) to minimize the potential impact of our necessary reliance on maternal questionnaires.

Although these potential limitations may lead to an underestimate of the prevalence of TS and CT in the ALSPAC sample, our results are consistent with other prevalence estimates of TS and CT in the general population, and this study is one of few to report the overlapping rates of co-occurring TS/CT, OCD, and ADHD in a population-based sample. Furthermore, this is the first study to examine the rates of TS and CT in the ALSPAC cohort, in which detailed longitudinal data about child development are available for more than 7,000 children. Through our strict inclusion/exclusion criteria and heterogeneity analyses, we believe that we have identified optimal disease definitions that both meet *DSM-IV* criteria and maintain face validity. Therefore, these definitions should prove extremely useful for future studies of TS and CT in this cohort.

## Figures and Tables

**TABLE S1:** Proportion of Children in the Avon Longitudinal Study of Parents and Children (ALSPAC) Cohort Whose Parents Endorsed the Presence of “Tics or Twitches” in Their Son/Daughter at Each Age-Specific Questionnaire

Child's Age at Time of Completion of Maternal Questionnaire	Children with Data Available at Age 13 Years	Children with Data Not Available at Age 13 Years	Overall Sample
1 Year 6 months	0.42% (26/6,155)	0.96% (41/4,280)	0.64% (67/10,435)
2 Years 6 months	0.54% (32/5,973)	0.73% (27/3,678)	0.61% (59/9,651)
3 Years 6 months	1.39% (84/6,048)	1.78% (62/3,476)	1.53% (146/9,524)
4 Years 9 months	0.80% (47/5,909)	1.25% (37/2,961)	0.95% (84/8,870)
5 Years 9 months	1.18% (68/5,740)	1.72% (41/2,379)	1.34% (109/8,119)
6 Years 5 months	1.37% (79/5,751)	2.22% (49/2,210)	1.61% (128/7,961)
7 Years 7 months	1.64% (93/5,681)	2.65% (51/1,922)	1.89% (144/7,603)
10 Years 8 months	2.29% (135/5,903)	3.32% (43/1,296)	2.47% (178/7,199)
13 Years	17.9% (1211/6,768)	NA	17.9% (1211/6,768)

Note: Rates of positive response to the single question about tics at each age are separated into children who had data available at age 13 years (eligible for inclusion in the current study) and children who were lost to follow-up before age 13 years (not eligible for inclusion in the current study due to absence of detailed tic questionnaire data only available at age 13). Rates of positive responses in the overall sample are also provided. These data do not include subjects with intellectual disability, autism, or IQ <80 as described in the main text.

**TABLE S2:** Response Rates in the Avon Longitudinal Study of Parents and Children (ALSPAC) Age 13 Mother-Completed Questionnaires to Each of the Detailed Questions about Specific Tics and Their Frequencies

Response	C1: Repeated Movements of Face and Head	C2: Repeated Movements of Neck, Shoulder, or Trunk	C4: Repeated Noises and Sounds in Past Year	Response	C6b: Frequency of These Habits
Definitely	327 (4.8%)	125 (1.9%)	308 (4.6%)	< Once a month	150 (2.2%)
Probably	303 (4.5%)	203 (3.0%)	479 (6.6%)	1–3 Times a month	164 (2.4%)
Not at all	6,063 (89.6%)	6,336 (93.6%)	5,910 (87.3%)	About once a week	151 (2.2%)
Missing	75 (1.1%)	104 (1.5%)	106 (1.6%)	> Once a week	501 (7.4%)
				Every day	667 (9.9%)
				Missing/NA	5,135 (75.9%)

Note: Exclusions for ID, autism, IQ <80 were conducted before these variables were tabulated.

**TABLE S3:** Prevalence Rates, Gender Ratios, and Rates of Co-occurring Obsessive-Compulsive Disorder (OCD) and Attention-Deficit/Hyperactivity Disorder (ADHD) in the Two Definitions of Chronic Motor Tics (CMT) and Chronic Vocal Tics (CVT)

	Prevalence Rate	Gender					OCD				ADHD			
M:F Ratio	Male % (n)	OR (95% CI)	*p* Value	I^2^ (*p*-het)	Total % (n)	OR (95% CI)	*p* Value	I^2^ (*p*-het)	Total % (n)	OR (95% CI)	*p* Value	I^2^ (*p*-het)
Controls		0.9:1	47 (2,833)				2 (122)				2 (106)			
CMT Narrow (n = 23)	0.3%	1.9:1	65 (15)	2.1 (0.8, 5.7)	.14	—	4 (1)	2.1 (0.1, 13.4)	.98	—	13 (3)	8.1 (1.5, 27.9)	.001	—
CMT Intermediate (n = 47)	0.2%	2.1:1	68 (32)	2.4 (1.2, 4.7)	.008	0%^a^ (*p* = .90)	9 (4)	4.4 (1.1, 12.3)	.012	75%^a^ (*p* = .045)	13 (6)	7.9 (2.7, 19.3)	<.001	0%^a^ (*p* = .73)
CVT Narrow (n = 12)	0.7%	2.0:1	67 (8)	2.2 (0.6, 10.1)	.30	—	17 (2)	9.4 (0.99, 44.6)	.013	—	17 (2)	10.8 (1.1, 51.5)	.006	—
CVT Intermediate (n = 25)	0.4%	3.2:1	76 (19)	3.50 (1.34, 10.73)	.008	0%^a^	12 (3)	6.4 (1.2, 21.7)	.006	0%^a^ (p = .83)	8 (2)	4.7 (0.53, 19.4)	.12	0%^a^ (*p* = .75)

Note: I^2^ is the percent variation due to heterogeneity rather than chance with I^2^ = 25%, 50%, and 75% suggesting low, moderate, and high heterogeneity, respectively.^1^ CI = confidence interval; M:F = male:female ratio; OR = odds ratio; *p*-het = *p* value for Cochran's Q heterogeneity test (*p* > 0.05 suggests lack of heterogeneity). Controls were defined as subjects who were eligible for analysis at age 13 years but did not meet any of the tic case definitions (see Figure 1).

a heterogeneity comparison between narrow and intermediate groups.

**TABLE S4:** Sensitivity Analysis of Tourette Syndrome (TS) and Chronic Tic (CT) Intermediate Definitions

	Prevalence Rate (95% CI)	Gender				OCD			ADHD		
M:F Ratio	Male % (n)	OR (95% CI)	*p* Value	Total % (n)	OR (95% CI)	*p* Value	Total % (n)	OR (95% CI)	*p* Value
Controls		0.9:1	47			2 (122)			2 (106)		
TS Intermediate (n = 50)	0.7% (0.5%–1.0%)	2.3:1	70 (35)	2.6 (1.4, 5.1)	.002	20 (10)	12.0 (5.2, 25.2)	<.001	18 (9)	12.2 (5.1, 26.2)	<.001
TS Intermediate Plus (n = 55)	0.8% (0.6%–1.1%)	2.2:1	69 (38)	2.5 (1.4, 4.7)	.002	19 (10)	10.6 (4.7, 22.0)	<.001	19 (10)	12.4 (5.4, 25.8)	<.001
CT Intermediate (n = 72)	1.1% (0.8%–1.3%)	2.4:1	71 (51)	2.7 (1.6, 4.7)	<.001	10 (7)	5.0 (1.9, 11.3)	<.001	11 (8)	6.8 (2.7, 14.6)	<.001
CT Intermediate Plus (n = 79)	1.2% (0.9%–1.5%)	2.2:1	68 (54)	2.4 (1.5, 4.0)	<.001	9 (7)	4.5 (1.7, 10.1)	<.001	10 (8)	6.1 (2.5, 13.2)	<.001

Note: TS Intermediate and CT Intermediate disease definitions were derived as described in the Method section of the text. A sensitivity analysis was performed by relaxing the tic frequency criterion to include children whose parents reported the presence of tics “about once a week” in addition to the two higher frequency responses “daily” and “more than once a week” (Table 1 and Table S2, available online). This analysis increased the TS Intermediate sample by five cases (TS Intermediate Plus) and the CT Intermediate sample by seven cases (CT Intermediate Plus). Prevalence rates based on the original definitions and relaxed definitions are provided along with gender ratios, and rates of co-occurring obsessive-compulsive disorder (OCD) and attention-deficit/hyperactivity disorder (ADHD). *p* values indicate comparisons between each definition and the control population. Controls were defined as subjects who were eligible for analysis at age 13 years but did not meet any of the tic case definitions (Figure 1). CI = confidence interval; OR = odds ratio.

**TABLE S5:** Comparison of Avon Longitudinal Study of Parents and Children (ALSPAC) Cohort Attendees of the Age 13 Assessment With Nonattendees

	Attendees n (%)	Nonattendees n (%)	*p* Value
Gender			
Male	3,351 (49.5)	3,743 (52.7)	<.001
Female	3,417 (50.5)	3,363 (47.3)
Maternal education			
< O'level	1,276 (20.2)	2,195 (39.7)	<.001
O ‘level	2,204 (34.9)	1,903 (34.4)
> O'level	2,834 (44.9)	1,437 (26.0)
Housing tenure			
Mortgaged/owned	5,301 (83.9)	4,131 (63.8)	<.001
Rented/other	1,015 (16.1)	2,346 (36.2)
Ethnicity of child			
White	5,982 (96.3)	4,972 (93.5)	<.001
Nonwhite	228 (3.7)	345 (6.5)
Maternal age (mean)	29.2 (4.6)	26.9 (5.1)	<.001

Note: Attendees at age 13 (n = 7,152) represent the subset of the ALSPAC sample whose mothers completed the Age 13 Questionnaire and thus were eligible for the present study. Nonattendees (n = 7,381) indicate subjects from the original ALSPAC birth cohort who were lost to follow-up before the Age 13 tic screening questionnaire.

**TABLE 1:** Definitions of Tourette Syndrome (TS) and Chronic Tics (CT) Based on Mother-Completed Questionnaires From the Avon Longitudinal Study of Parents and Children (ALSPAC)

	TS	CT
**Narrow Definition**	1) Motor and Vocal Tics: Response of “Definitely” to motor AND vocal tic questions at Age 13	1) Motor OR Vocal Tics: Response of “Definitely” to motor OR vocal tic questions (not both) at Age 13
2) Frequency: Daily	2) Frequency: Daily
3) Chronicity: Positive response to tic screening question at 1 other time point	3) Chronicity: Positive response to tic screening question at 1 other time point 4) Exclusions: IQ <80 or autism
4) Exclusions: IQ <80 or autism	
**Intermediate Definition** (same as Narrow except “Probably” allowed in response to tic questions and frequency expanded to include daily-weekly)	1) Motor AND Vocal Tics: Response of “Definitely” or “Probably” to motor AND vocal tic questions at age 13	1) Motor OR Vocal Tics: Response of “Definitely” or “Probably” to motor OR vocal tic questions at age 13
2) Frequency: Daily or >once per week	2) Frequency: Daily or >once per week
3) and 4) Chronicity and Exclusions: Same criteria as for Narrow Definition	3) and 4) Chronicity and Exclusions: Same criteria as for Narrow Definition
**Broad Definition** (Relaxed to remove chronicity requirements; designed to capture subjects with onset after Age 10 or missed in early screens)	1) Motor AND Vocal Tics: Response of “Definitely” or “Probably” to motor AND vocal tic questions at age 13	1) Motor OR Vocal Tics: Response of “Definitely” or “Probably” to motor AND vocal tic questions at age 13
2) Frequency: Daily or >once per week	2) Frequency: Daily or >once per week
3) Chronicity: No chronicity requirement	3) Chronicity: No chronicity requirement
4) Exclusions: IQ <80 or autism	4) Exclusions: IQ <80 or autism

Note: Diagnoses of probable TS and CT were derived based on three levels of diagnostic stringency (Narrow, Intermediate, and Broad) to define TS and CT using *DSM-IV-TR* criteria. Specific tic symptom questions are provided in the text and on the ALSPAC Web site (http://www.bristol.ac.uk/alspac/sci-com/quests/).

**TABLE 2:** Prevalence of Tourette Syndrome (TS) and Chronic Tics (CT) Using Narrow, Intermediate and Broad Definitions

Definition	TS			CT		
n	Prevalence, %	95% CI	n	Prevalence, %	95% CI
**Narrow**	23	0.3	0.2–0.5	35	0.5	0.4–0.7
**Intermediate**	50	0.7	0.5–1.0	72	1.1	0.8–1.3
**Broad**	217	3.2	2.8–3.7	583	8.6	7.9–9.3

Note: Prevalence rates were calculated from the number of children whose mothers completed the age 13 questionnaire and who did not have autism or intellectual disability (n = 6,768). CI = confidence interval.

**TABLE 3:** Gender Ratios and Rates of Co-occurring Obsessive-Compulsive Disorder and Attention-Deficit/Hyperactivity Disorder Across the Two Definitions of Tourette Syndrome and Chronic Tics

	Gender					OCD				ADHD			
M:F Ratio	Male % (n)	OR (95% CI)	*p*-Value	I^2^ (p-het)	Total % (n)	OR (95% CI)	*p*-Value	I^2^ (p-het)	Total % (n)	OR (95% CI)	*p*-Value	I^2^ (p-het)
Controls	0.9:1	47 (2,833)				2 (122)				2 (106)			
TS Narrow	3.6:1	78 (18)	4.0 (1.4, 13.7)	0.006	—	22 (5)	13.0 (3.7, 37.1)	<0.001	—	17 (4)	11.4 (2.8, 35.0)	<0.001	—
TS Intermediate	2.3:1	70 (35)	2.6 (1.4, 5.1)	0.002	32.8%^a^ (*p* = 0.22)	20 (10)	12.0 (5.2, 25.2)	<0.001	0%^a^ (*p* = 0.83)	18 (9)	12.2 (5.1, 26.2)	<0.001	0%^a^ (*p* = 0.87)
CT Narrow	1.9:1	66 (23)	2.1 (1.0, 4.7)	0.047	—	9 (3)	4.4 (0.9, 14.3)	0.039	—	14 (5)	9.0 (2.7, 24.1)	<0.001	—
CT Intermediate	2.4:1	71 (51)	2.7 (1.6, 4.7)	<0.001	0%^a^ (*p* = 0.64)	10 (7)	5.0 (1.9, 11.3)	<0.001	0%^a^ (*p* = 0.86)	11 (8)	6.8 (2.7, 14.6)	<0.001	2.5%^a^ (*p* = 0.31)

Note: I^2^ is the percent variation due to heterogeneity rather than chance with I^2^=25%, 50%, and 75% suggesting low, moderate, and high heterogeneity, respectively.^26^ ADHD = attention-deficit/hyperactivity disorder; M:F = male:female ratio; OCD = obsessive compulsive disorder; OR = odds ratio; *p*-het = *p* value for Cochran's Q heterogeneity test (*p* >0.05 suggests lack of heterogeneity).

a heterogeneity comparison between narrow and intermediate groups. Controls were defined as subjects who were eligible for analysis at age 13 years but did not meet any of the tic case definitions.
